# Assessing the accuracy of magnetic resonance imaging in identifying early rectal cancers suitable for endoscopic intermuscular dissection

**DOI:** 10.1055/a-2621-2515

**Published:** 2025-07-14

**Authors:** Lisa van der Schee, Rachel Carten, Sander C. Albers, Janneke van den Bergh, Manon N. G. J. A. Braat, Arnold C. Goede, Sabrine Kol, Miangela M. Lacle, Banafsche Mearadji, Shira I. Moos, Irene M. Nota, Nicky H. G. M. Peters, Jip F. Prince, Jorik J. Reimerink, Arantza Fariña Sarasqueta, Jeanette van Vooren, Jan Hein T. M. van Waesberghe, Barbara A. J. Bastiaansen, Frank P. Vleggaar, Leon M. G. Moons, Karin Horsthuis, Gina Brown

**Affiliations:** 1 8124 Department of Gastroenterology and Hepatology, University Medical Centre Utrecht, Utrecht, Netherlands; 2 Department of Pathology, University Medical Center Utrecht, Utrecht, Netherlands; 3 1174 Department of Colorectal Surgery, Buckinghamshire Healthcare NHS Trust, Aylesbury, United Kingdom of Great Britain and Northern Ireland; 4 Department of Surgery and Cancer, Imperial College London, London, United Kingdom of Great Britain and Northern Ireland; 5 Department of Gastroenterology and Hepatology, Amsterdam University Medical Centers, Amsterdam, Netherlands; 6 Department of Radiology, Amsterdam University Medical Centers, Amsterdam, Netherlands; 7 Department of Radiology, University Medical Center Utrecht, Utrecht, Netherlands; 8 Department of Radiology, Maastricht University Medical Center, Maastricht, Netherlands; 9 Department of Pathology, Amsterdam University Medical Centers, Amsterdam, Netherlands; 10 Department of Gastroenterology and Hepatology, University Medical Center Utrecht, Utrecht, Netherlands; 11 4615 Department of Radiology, Imperial College London, London, United Kingdom of Great Britain and Northern Ireland

## Abstract

**Background:**

Selection of rectal cancers suitable for endoscopic intermuscular dissection (EID) is challenging. We aimed to evaluate whether identification of ≥1 mm of preserved muscularis propria on magnetic resonance imaging (MRI), using a systematic reporting tool (mrSRT), can identify rectal cancers suitable for EID.

**Methods:**

An expert radiologist trained 12 study radiologists in the use of the mrSRT. The radiologists then assessed a retrospective series of MRIs from 269 consecutive patients with suspected deep submucosal invasive rectal cancer. The primary objective was to determine the diagnostic accuracy of ≥1 mm of preserved muscularis propria on MRI for selecting cases that can be resected with clear margins using EID (invasion limited to the circular muscularis propria [≤pT2circ]). Diagnostic accuracy was calculated for the expert radiologist, study radiologists, and a consensus diagnosis.

**Results:**

After applying the inclusion and exclusion criteria, 244 patient scans were included in the analysis. Histological classification confirmed 18 lesions (7.4%) were noninvasive, 109 (44.7%) were pT1, 56 (23.0%) were pT2circ, 21 (8.6%) were pT2long, 39 (16.0%) were pT3, and 1 (0.4%) was pT4. The overall diagnostic accuracy of ≥1 mm of preserved muscularis propria as a criterion for selection was 80.7% (95%CI 75.2–85.5) for the expert radiologist, 77.5% (95%CI 71.7–82.5) for the trained study radiologists, and 81.6% (95%CI 76.1–86.2) for a consensus diagnosis.

**Conclusion:**

Use of mrSRT to identify ≥1 mm of preserved muscularis propria on MRI allowed radiologists to assist in appropriate case selection for EID.

## Graphical Abstract

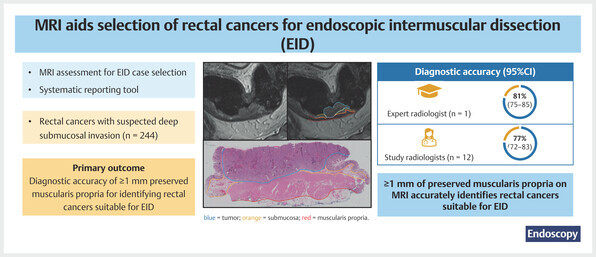

## Introduction

The introduction and expansion of colorectal cancer screening programs has led to an increase in the detection of early-stage (pT1–2N0) rectal cancer in recent years 1 2 . Patients who are diagnosed with early rectal cancer (ERC) may be eligible for rectal preservation.

Rectal-preserving strategies offer several advantages over traditional total mesorectal excision (TME) surgery, as they allow treatment of the primary tumor while preserving normal rectal anatomy. TME surgery carries a significant risk of perioperative morbidity and mortality 3 , and can result in long-term undesirable effects on a patient’s quality of life, such as urinary, sexual, and bowel dysfunction, including low anterior resection syndrome. In many cases, it also necessitates the formation of a temporary or permanent stoma 4 . Therefore, increasing the number of patients who can consider a rectal-preserving strategy while avoiding the perioperative risks and longer-term negative impact on quality of life of major TME resection would be desirable 5 .

The recent introduction of endoscopic intermuscular dissection (EID) enables complete (R0) resection of deep submucosal invasive (T1b) rectal cancers and even superficial T2 rectal cancers, where invasion is limited to the circular layer of the muscularis propria (pT2 _circ_ ) 6 . Given that deep submucosal invasion is no longer regarded as an independent risk factor for lymph node metastasis, EID can provide definitive treatment for patients with T1b rectal cancer where deep submucosal invasion is the sole risk factor 7 . In the case of patients with pT2 _circ_ rectal cancer, an R0 resection using EID could permit curative treatment, allowing patient choice to pursue rectal preservation with adjuvant (chemo)radiotherapy and/or active surveillance with early salvage surgery if required 8 .

However, selection of appropriate cases for EID remains a significant challenge 6 . While endoscopic optical diagnosis can differentiate between benign and invasive lesions, where deep submucosal invasion is suspected based on endoscopic features (Japan Narrow-band imaging [NBI] Expert Team [JNET] 3/Hiroshima C2-C3), there is currently no reliable method to accurately differentiate between T1b-T3 rectal cancers. Historically, the accuracy of magnetic resonance imaging (MRI) for ERC staging has been poor, with considerable rates of misdiagnosis 9 10 11 . Furthermore, it is evident in both clinical practice and most published studies that there is often no attempt to distinguish between T1 and T2 stages. This may be due to a current lack of evidence that this is possible, despite the growing clinical importance of distinguishing between T1 and T2 tumors. To enhance the efficacy of EID, improvements in pretherapeutic diagnosis are required.

In a previous study, the potential for more precise MRI staging for ERC was demonstrated, with an 89% accuracy in detecting tumors suitable for local excision using a systematic reporting tool (mrSRT). The mrSRT identifies and differentiates between the visible layers of the rectal wall, allowing the degree of preservation in each layer to be measured as part of the staging assessment 12 . However, its diagnostic accuracy has not yet been assessed in a larger cohort, nor has its applicability been investigated in a broader group of radiologists.

The primary aim of the present study was to evaluate the diagnostic accuracy of MRI in identifying ≥1 mm of preserved muscularis propria, thereby distinguishing between tumors that can be successfully resected with clear margins using EID, from those that cannot. The evaluation was done for both an expert radiologist and a cohort of radiologists newly trained in the use of the mrSRT. A secondary aim was to investigate whether any patient-related, tumor-specific, or scan-related factors were associated with inaccurate MRI-based T staging.

## Methods

### Study sample and design

**Fig. 1:**
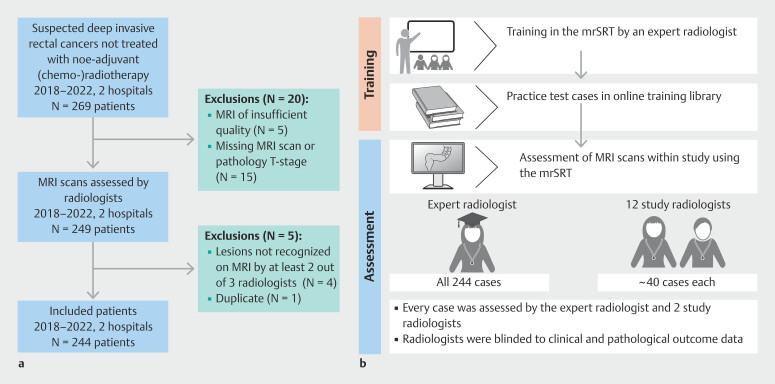
Study flow charts. **a** Patients. **b** Procedures

A retrospective cohort of 269 consecutive adult patients with rectal lesions suspected of (at least) deep submucosal invasion at initial endoscopic assessment between 1 January 2018 and 1 March 2022 was collated from two large academic hospitals in the Netherlands ( Fig. 1 **a** ). All patients had subsequent primary excision of the lesion to ensure that a final pathological T stage was confirmed for each case. Patients underwent a variety of excision methods, including local endoscopic excision (e.g. endoscopic submucosal dissection [ESD], EID), local surgical excision (e.g. transanal minimally invasive surgery, transanal endoscopic microsurgery), and radical surgical excision (TME surgery).

**Fig. 2:**
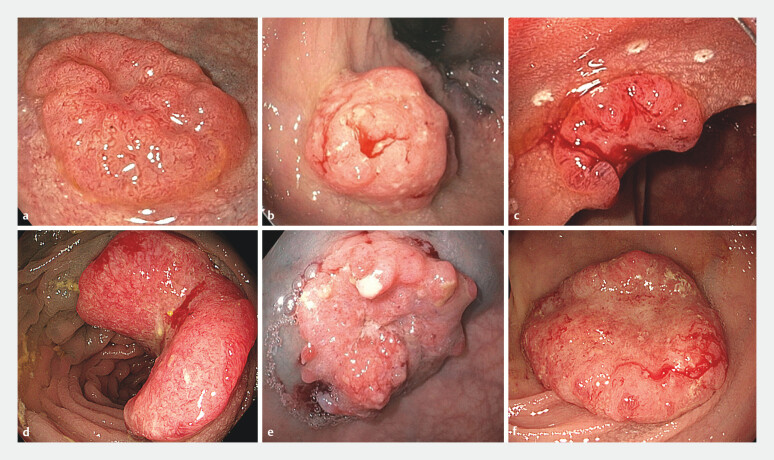
Examples of tumors included in the study with features suspicious for deep submucosal invasion. Final histology results showed: **a** adenoma with high grade dysplasia; **b** T1 sm1 cancer; **c** T1 sm1 cancer; **d** T2 cancer limited to the circular muscularis propria (T2 _circ_ ); **e** T2 _circ_ ; **f** T2 _circ_ .

The endoscopic optical features of (at least) deep submucosal invasion were defined as either Hiroshima C2-C3, JNET 3, or NBI International Colorectal Endoscopic classification 3 with NBI, or Kudo Vn pit pattern with chromoendoscopy 13 14 15 16 . In the absence of a description of these features in the endoscopy report, the depth of invasion was based on the narrative description provided by the endoscopist, indicating suspicion of deep invasion and/or the inability to resect the polyp through local excision with ESD, EID, or transanal minimally invasive surgery. Six examples of tumors with suspected deep submucosal invasion are presented in Fig. 2 .

To be eligible for inclusion in the study, patients had to meet all of the following inclusion criteria: ≥18 years of age; rectal lesion displaying features of (at least) deep submucosal invasion, with rectum being defined as located at or below the sigmoid take-off on MRI; pre-therapeutic MRI had been performed; no neoadjuvant (chemo)radiotherapy was given prior to excision; final histology reported an adenomatous polyp or adenocarcinoma (e.g. excluding neuroendocrine cancers); and definite pathological T stage was known. Patients were excluded from analysis if MRI scans were nondiagnostic due to severe artifacts (e.g. extreme motion or metal-induced artifacts). After assessment by all radiologists, five cases were excluded from further analyses: one was a duplicate case and in four cases the lesion could not be recognized by at least two out of three assessing radiologists ( Fig. 1 **a** ).

This study was approved by the Medical Ethics Review Committee of the University Medical Center Utrecht (reference number 22–794) on August 9, 2022, and conducted in accordance with the Helsinki Declaration.

### Study variables

Patient characteristics, endoscopy results, initial radiology reports, treatment details, and pathology results were obtained by extracting data from individual electronic medical records. The size of the lesion and its height within the rectum, measured from the dentate line, were gathered from the initial endoscopy report and arbitrarily categorized as lower (0–5 cm), mid (6–10 cm), or upper rectum (11 cm to sigmoid take-off). In cases where the endoscopy report did not provide this information, measurements of lesion size and distance from the anorectal junction were taken from the original MRI report from the participating hospital (i.e. the MRI report that was used in clinical decision making, hereafter referred to as “original MRI report”). The pretherapeutic staging diagnosis was derived from the original MRI report. In many cases, the cT stage was classified as cT1–2, as the distinction between cT1 and cT2 is not current standard clinical practice. Histology reports were used to obtain the definite pathological T stage for all pT1 rectal cancers, with depth of invasion subclassified according to the Kikuchi classification 17 . All specimens reported as pT2 in original histology reports were reassessed by experienced histopathologists (M.M.L. and A.F.S.) to determine whether invasion was confined to the inner circular muscle layer (pT2 _circ_ ) or extended into the deep longitudinal muscle layer (pT2 _long_ ), as this is not currently reported as standard.

A detailed description of other variables included in the study is presented in the online-only Supplementary Methods.

### Participating radiologists and radiologist training

A total of 14 Dutch radiologists were recruited to participate in the study. All were subspecialist abdominal radiologists with an interest in rectal MRI ( **Table 1s** ). The study radiologists attended one of two half-day online training sessions conducted by the expert radiologist (G.B.), during which they were taught how to assess for invasion in each of the layers of the rectal wall on MRI, and how to classify their scan findings ( Table 1 ). A summary of the techniques taught can be found in the Supplementary material.

**Table:** **Table 1** Classification of magnetic resonance imaging (MRI) findings according to the systematic MRI reporting tool.

MRI assessment features	MRI T staging
No macroscopic evidence of submucosal invasion	T0 (benign)
≥1 mm macroscopically visible spared submucosa and fully intact muscularis propria	T1sm1/sm2
<1 mm submucosa preserved with ≥1 mm macroscopically intact fibers of muscularis propria	T1sm3/T2 _circ_
<1 mm submucosa preserved with <1 mm macroscopically intact fibers of muscularis propria, or invasion through muscularis propria	≥T2 _long_
T2 _circ_ , pT2 invasion limited to the circular layer of the muscularis propria; T2 _long_ , pT2 invasion into the longitudinal layer of the muscularis propria.	

After the training session, radiologists had access to an online library where they could practice, and two further feedback sessions were held, during which the expert radiologist addressed queries raised by the radiologists, prior to the study commencing ( Fig. 1 **b** ).

### Assessment of MRI scans

MRI scans obtained for the included patients were pseudonymized prior to being uploaded onto a secure cloud-based online picture archiving and communication system viewing platform, which radiologists used to review scans for the study. The expert radiologist (G.B.) assessed the entire patient/scan cohort. Two study radiologists withdrew from the study prior to starting any reporting. The remaining 12 radiologists were assigned 36–45 scans each, ensuring that all scans were reported by at least two study radiologists: a first reporter and a second reporter ( Fig. 1 **b** ). The allocation of scans to study radiologists was conducted by a researcher who was blinded to patient data. This researcher ensured that a mixture of scans from different sites and dates were allocated to each of the study radiologists. The radiologists then completed assessments for cases as first reporter. Once complete, scans were then reallocated to a different radiologist in a similar blinded manner to ensure a variety of reporting combinations from across the radiologist and patient cohorts. Radiologists then completed assessments as a second reporter.

Radiologists were asked to record the following parameters in their assessment: distance of the lesion from the anorectal junction; size and morphology of the lesion; rectal wall preservation level; radiological T and N stage; presence/absence of extramural vascular invasion and tumor deposits; circumferential resection margin status; and for study radiologists only, a self-assessment of their confidence in reporting T stage, N stage, extramural vascular invasion, and tumor deposits, given as either >70% or ≥70%. The expert radiologist (G.B.) also provided a subjective assessment of the technical quality of each scan.

Radiologists entered their staging assessment via an electronic data capture system. Radiologists were blinded to assessments made by other radiologists and to all clinical and pathological outcome data matched to the MRI scans.

### End points

**Fig. 3:**
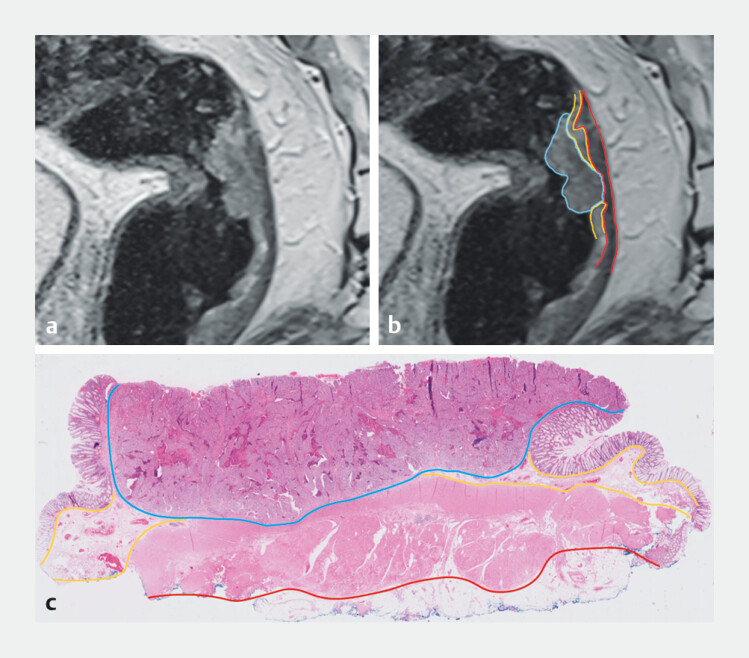
An example of a pT2 rectal cancer, limited to the circular layer of the muscularis propria. **a** Sagittal sequence showing the center of the tumor. **b** Loss of visible submucosa (yellow) with >1 mm preserved muscularis propria (red) in the central portion of this early T2 tumor (blue). **c** Histological examination confirmed the presence of a pT2 rectal cancer, with invasion limited to the circular layer of the muscularis propria.

The primary objective was to assess the diagnostic accuracy of ≥1 mm of preserved muscularis propria on MRI, assessed using the mrSRT, for identifying rectal tumors with, at most, a pT2 _circ_ , indicating that they could be removed with clear (R0) margins using EID. Illustrative cases are presented in Fig. 3 and **Fig. 1s** .

The secondary end points were the sensitivity, specificity, positive likelihood ratio (PLR), and negative likelihood ratio (NLR) of measuring ≥1 mm of preserved muscularis propria on MRI using the mrSRT, for the identification of ≤pT2 _circ_ rectal tumors. Additionally, we aimed to assess whether a new cohort of radiologists could be trained to accurately use the mrSRT, including an evaluation of interobserver agreement and interobserver reliability between the expert and trained study radiologists. Furthermore, we investigated whether image quality (high resolution vs. suboptimal resolution), patient-related, or lesion-related factors were associated with misdiagnosis by MRI. An MRI scan was classified as “high resolution” if all sequences had a voxel size of <1.1 mm and a slice thickness of ≤3 mm 18 19 . If one or more sequences did not meet these criteria, the scan was categorized as having “suboptimal resolution.”

The primary and secondary objectives were evaluated for the expert radiologist, the trained study radiologists, and a consensus diagnosis. To evaluate the combined performance of the trained study radiologists, their “first reporter” assessments were utilized. In order to evaluate an individual radiologist’s performance, both assessments made as first and second reporters were included. The consensus diagnosis was based on the MRI staging assessment of the two study radiologists (first and second reporters) who had independently reported the case and assessed it as locally resectable (≥1 mm preserved muscularis propria) or not locally resectable (<1 mm preserved muscularis propria) according to the mrSRT. In cases of discordance between the study radiologists, the assessment of the expert radiologist was used to reach a final consensus diagnosis.

### Statistical analysis

We used descriptive statistics to present baseline characteristics. Categorical data were expressed as frequencies with percentages, and non-normally distributed continuous data were described using the median with corresponding interquartile range (IQR). A rectal cancer was considered locally resectable in the intermuscular plane when the pathology results confirmed a superficial pT2 rectal cancer (limited to the circular muscularis propria). We calculated the diagnostic accuracy, sensitivity, specificity, PLR, and NLR for identification of ≥1 mm of preserved muscularis propria on MRI using the mrSRT in identifying tumors suitable for local excision with corresponding 95%CIs for the expert radiologist, the study radiologists, and the consensus diagnosis. Interobserver agreement of MRI diagnosis (i.e. suitability for local excision in the intermuscular plane) among the expert and study radiologists was evaluated through absolute percentages of agreement, whereas Fleiss kappa was used to assess interobserver reliability. Finally, through univariable logistic regression analyses, we explored the potential association between clinical characteristics, tumor-related factors, and the expert radiologists’ subjective assessment of scan quality, with MRI misdiagnosis.

## Results

Of 269 identified patients, 244 were eligible for inclusion in the final analyses ( Fig. 1 **a** ). Details of the patient and lesion characteristics are provided in Table 2 . According to our predefined criteria, 183/244 rectal lesions (75.0%) did not invade beyond the circular layer of the muscularis propria based on histopathological evaluation and would therefore have been suitable for local excision in the intermuscular plane.

**Table:** **Table 2** Characteristics of the 244 patients included in the study.

Variable	N = 244
Age, median (IQR), years	67 (13)
Sex, n (%)	
Female	71 (29.1)
Male	173 (70.9)
ASA classification, n (%)	
I	44 (18.0)
II	168 (68.9)
III	32 (13.1)
Lesion location ^1^ , n (%)	
Upper rectum (11–15 cm from anal verge)	45 (18.9)
Mid rectum (6–10 cm from anal verge)	55 (23.1)
Lower rectum (0–5 cm from anal verge)	138 (58.0)
Missing	6
Lesion size ^1^ , median (IQR), mm	25 (20)
cT stage original MRI, n (%)	
No invasion	2 (0.8)
cT1	7 (2.9)
cT1–2	141 (58.5)
cT2	40 (16.6)
cT3	50 (20.7)
cT3a	15
cT3b	19
cT3c	5
cT3 (substage not specified)	11
cT4a	1 (0.4)
Missing	3
pT stage, n (%)	
Adenoma	18 (7.4)
pT1	109 (44.7)
sm1	9
sm2	32
sm3	50
Unknown sm substage ^2^	18
pT2	77 (31.6)
Circular muscularis propria	56
Longitudinal muscularis propria	21
pT3	39 (16.0)
pT4	1 (0.4)
Treatment strategy, n (%)	
Local excision only	117 (48.0)
Local excision + completion TME	62 (25.4)
Primary TME	65 (26.6)
ASA, American Society of Anesthesiologists; IQR, interquartile range; MRI, magnetic resonance imaging; TME, total mesorectal excision. ^1^ Location and size of the lesion as assessed during endoscopy, and in case of missing data, the data from the original MRI report was used. ^2^ Missing histopathology data on the specific depth of tumor invasion into the submucosa because of absence of muscularis propria in the resection specimen.	

### Diagnostic accuracy

#### Expert radiologist

The expert radiologist correctly classified 197 of 244 rectal lesions as being locally resectable or not, yielding a diagnostic accuracy of 80.7% (95%CI 75.2%–85.5%). The sensitivity, specificity, PLR, and NLR of the reporting approach were 84.7% (95%CI 78.7%–89.6%), 68.9% (95%CI 55.7%–80.1%), 2.72 (95%CI 1.86–3.97), and 0.22 (95%CI 0.15–0.33), respectively ( Table 3 ).

**Table:** **Table 3** Magnetic resonance imaging accuracy of expert radiologist’s assessment in identifying rectal lesions suitable for local excision in the intermuscular plane of the muscularis propria.

	pT stage		Total
	≤T2 circular muscularis propria	≥T2 longitudinal muscularis propria	
MRI staging			
≥1 mm of preserved muscularis propria	155	19	174
<1 mm of preserved muscularis propria	28	42	70
Total	183	61	244
MRI, magnetic resonance imaging.			

#### Study radiologists

A total of 12 radiologists from three academic hospitals participated in the study. The median number of years of experience as an abdominal radiologist was 5.5 (IQR 8.75), and all reported at least 100 rectal cancer MRI scans during the course of their careers ( **Table 1s** ).

Together, the 12 trained study radiologists correctly classified 189 of 244 rectal lesions as being locally resectable or not, yielding a diagnostic accuracy of 77.5% (95%CI 71.7%–82.5%). The sensitivity, specificity, PLR, and NLR of the reporting approach were 78.1% (95%CI 71.4%–83.9%), 75.4% (95%CI 62.7%–88.5%), 3.18 (95%CI 2.03–4.96), and 0.29 (95%CI 0.21–0.39), respectively ( Table 4 ).

**Table:** **Table 4** Magnetic resonance imaging accuracy of trained study radiologists’ assessments in identifying rectal lesions suitable for local excision in the intermuscular plane of the muscularis propria.

	pT stage		Total
	≤T2 circular muscularis propria	≥T2 longitudinal muscularis propria	
MRI staging			
≥1 mm of preserved muscularis propria	143	15	158
<1 mm of preserved muscularis propria	40	46	86
Total	183	61	244
MRI, magnetic resonance imaging.			

The median accuracy score for all individual study radiologists, including cases assessed as both first and second reporter, was 77.6% (IQR 10.7%). Individual diagnostic accuracy rates ranged from 64.3% to 94.9% ( **Table 2s** ). No evidence of a learning curve was observed, as there was no statistically significant difference in diagnostic accuracy between cases assessed as a first or second reporter.

Radiologists participating in the study were asked to indicate whether their confidence in the T-stage diagnosis was below or above 70%. In 61.8% of cases, radiologists reported confidence levels below 70%. In these instances, the diagnostic accuracy was 75.5%, compared with 80.7% when confidence levels exceeded 70%. Notably, specificity increased from 69.7% to 82.1% when radiologists were more confident in their assessments, whereas sensitivity exhibited only a marginal difference (77.1% vs. 80.0%).

#### Consensus diagnosis

In 186 of 244 cases (76.2%), the study radiologists agreed on whether or not a lesion was suitable for local excision in the intermuscular plane. For the remaining 58 cases (23.8%) without agreement, the expert radiologist’s assessment was used to establish a final consensus diagnosis.

The diagnostic accuracy of the consensus diagnosis was 81.6% (95%CI 76.1%–86.2%). The sensitivity, specificity, PLR, and NLR were 84.2% (95%CI 78.0%–89.1%), 73.8% (95%CI 60.9%–84.2%), 3.21 (95%CI 2.10–4.91), and 0.21 (95%CI 0.15–0.31), respectively.

### Interobserver agreement and reliability

Complete agreement on local resectability in the intermuscular plane among all three radiologists was observed in 63.1% of all cases. Interobserver reliability was moderate (Fleiss κ 0.45).

### Factors associated with MRI misdiagnosis

#### Quality of the scans

The technical details of all MRI sequences in the study can be found in **Table 3s** . The expert radiologist subjectively assessed 17/244 scans (7.0%) as being of poor quality. Although not statistically significant, misdiagnosis was higher in cases with poor-quality scans compared with those of acceptable quality (35.3% vs. 18.0%; odds ratio 2.47, 95%CI 0.81–6.90). For cases with a high-resolution scan (voxel <1.1 and a slice thickness of ≤3 mm), diagnostic accuracy of the expert radiologist was 88.9% (95%CI 76.0%–96.3%), whereas this was 78.9% (95%CI 72.6%–84.4%) for cases with an MRI of suboptimal resolution.

#### Other factors

The analyses did not reveal any statistically significant associations between patient age, sex, or body mass index, nor size or location of the rectal lesion (upper, mid, or lower rectum) and the occurrence of misdiagnosis on MRI for the expert radiologist, study radiologists, or consensus diagnosis, although statistical power was limited ( **Table 4s** ).

## Discussion

Rectal MRI scans of patients with suspected deep submucosal invasive rectal cancer were reassessed by an expert radiologist and 12 trained study radiologists using the mrSRT. The study aimed to evaluate the diagnostic accuracy of measuring ≥1 mm of preserved muscularis propria on MRI as a reliable criterion for selecting patients for EID. Our results demonstrated an overall accuracy rate of 80.7% (95%CI 75.2–85.5) for the expert radiologist and 77.5% (95%CI 71.7–82.5) for the trained study radiologists. These results indicate that MRI can facilitate the accurate selection of candidates for EID, and that the method can be employed by radiologists with varying levels of experience and expertise after limited specialist training. We hypothesize that broader implementation of the mrSRT could increase the proportion of patients being offered the option of local excision for suspected rectal cancer and improve the R0 resection rates by facilitating identification of the optimal dissection plane.

Previously, deep submucosal invasion was widely regarded as a risk factor for lymph node metastasis in T1 colorectal cancer and, therefore, as an indication for completion surgery if demonstrated after local excision. This has been challenged in recent years, with multiple publications demonstrating the low risk of lymph node metastasis associated with deep submucosal invasion when other histological risk factors are absent 7 20 21 . As a result, a substantial subgroup of deep submucosal invasive T1 colorectal cancers can now potentially be treated curatively with local excision alone. For treatment of deep submucosal invasive cancers in the rectum, EID offers several advantages over other local resection techniques. Although ESD is effective for removing superficial T1 rectal cancers, the R0 resection rates drop to 35%–65% if deep submucosal invasion is present, whereas high R0 resection rates of 90% can be achieved with EID 6 22 23 . Transanal minimally invasive surgery can achieve similar R0 resection rates to EID; however, this often involves full-thickness resection, which could increase the risk of low anterior resection syndrome and potentially complicate subsequent TME surgery if indicated 24 25 26 . As the integrity of the rectal wall is generally not compromised with EID, we anticipate that these risks would be minimized following intermuscular dissection, and this is currently under investigation in a prospective trial (Dutch Trial Register NL8409).

This study demonstrates that identification of ≥1 mm of preserved muscularis propria on MRI can aid in distinguishing between tumors that can be successfully removed with clear deep margins with EID, and those that cannot. However there remains a possibility of both over- and understaging of lesions. Our reported sensitivity of 85% indicates that 15% of pT1 and superficial pT2 cancers could be overstaged and therefore miss the opportunity for rectal-preserving local excision. Of these, only 12 were pT1 rectal cancers, demonstrating an 89% sensitivity for the T1 subgroup. As many international guidelines still recommend surgical resection as the primary treatment for lesions suspected of having deep submucosal invasion 16 27 28 , it can be argued that pursuing a rectal-preserving strategy in 85% of such lesions already represents a major step forward. Our reported specificity was 69%, indicating that 31% of tumors with at least deep T2 invasion, might be understaged on MRI and incorrectly considered suitable for local excision. While we acknowledge that it would be preferable for this percentage to be lower, patients could still undergo a formal mesorectal resection after EID with no compromise expected.

We believe that there are several potential factors that could enhance the reported diagnostic accuracy and interobserver reliability of the mrSRT. First, implementing a standardized MRI protocol specifically designed to produce high-quality rectal MRI scan images would be beneficial. The retrospective nature of this study led to the utilization of MRI scans that were not conducted according to a predefined protocol. Consequently, there was variability in the technical quality of the scans, and a considerable percentage of the axial and coronal scans (48.4%) did not meet the slice thickness of ≤3 mm as recommended by the European Society of Gastrointestinal and Abdominal Radiology (ESGAR) 18 . While the inclusion of MRI scans from a broad range of different healthcare settings increases the generalizability of our findings to a wider patient population, standardization of MRI protocols to ensure consistent high-quality imaging could improve the radiologists’ assessments 29 . This is supported in the current study by the finding that the diagnostic accuracy of the expert radiologist was 89% when all sequences of the MRI scan met the criteria for a high-resolution scan (voxel <1.1 and a slice thickness of ≤3 mm), compared with 79% when these criteria were not met. Second, the addition of endorectal ultrasound (ERUS) to the pretherapeutic work-up may add diagnostic value. In some published studies, ERUS outperforms MRI in staging ERC 30 31 . However, to the best of our knowledge, no studies have specifically compared the accuracy of T staging in the subpopulation of patients with suspected deep submucosal invasive rectal cancer. Although ESGAR advocates for the use of ERUS in the staging of ERC considered for local excision, several studies have demonstrated a lack of integration of ERUS in everyday practice for the standard evaluation of ERC, with an uptake of only 4.5%–33% 18 32 33 . This may indicate that even when high accuracy levels of T staging could be achieved with ERUS, experience in this method might not yet be sufficient for its universal adoption in the evaluation of ERC. Nevertheless, it would be worthwhile testing whether there is an additive value in a multimodal staging approach for ERC, combining optical endoscopic findings, MRI staging with mrSRT, with or without ERUS staging 34 . By optimizing training, establishing standardized technical protocols, and utilizing consistent assessment methods for all modalities involved, we could further improve the selection of the most appropriate treatment strategy for patients with ERC, including the determination of the plane of dissection when local excision is considered feasible.

Our study has a few limitations that should be addressed. First, the retrospective nature of the study resulted in considerable variability in MRI scan quality. While this variability enhances the generalizability of the findings by reflecting routine clinical practice, it may have influenced the results and contributed to our inability to identify factors associated with misdiagnosis. Second, the study radiologists expressed a lack of confidence in their staging assessments; in 62% of cases they reported being “less than 70% confident” about the exact T stage, despite their results being comparable to the expert radiologist. To facilitate successful implementation, training may need to be intensified, incorporating more frequent and individualized feedback sessions 35 . Furthermore, although the accuracy rates achieved in the study are promising and exceed those reported in earlier studies, there remains room for improvement in terms of specificity, inter-rater reliability, and accuracy rates among radiologists. It is anticipated that optimizing training protocols and standardizing MRI protocols to ensure high-quality imaging will further enhance diagnostic performance. Additionally, it is important to note that our study cohort was selected based on the endoscopic appearance of suspected deep submucosal invasion, excluding lesions that were either clearly benign or advanced malignancies. In contrast, previous studies did not apply such stringent selection criteria. Consequently, the diagnostic accuracy of 80.7% observed in this study represents a significant advancement compared with previously reported results (53.8% and 67.9%) 9 10 11 . Finally, it is worth noting that while the participating radiologists in our study had varying levels of experience, they were all affiliated with academic hospitals. Therefore, it might be necessary to evaluate the diagnostic accuracy of radiologists working in nonacademic settings.

In conclusion, our study demonstrated the value of the mrSRT in identifying candidates with ERC suitable for local excision with EID, irrespective of the radiologists’ experience or expertise. Prospective evaluation and further research are required to assess the impact of the mrSRT on clinical decision making and to confirm its accuracy in clinical practice.

## Supplementary Material

Supplementary Material Zusatzmaterial
