# Supplementary material for: Assessing the accuracy of magnetic resonance imaging in identifying early rectal cancers suitable for endoscopic intermuscular dissection
Source: Endoscopy. 2025 Jul 14;57(12):1376–85. doi: 10.1055/a-2621-2515 (PMC12668270; doi:10.1055/a-2621-2515)

Thieme

SUPPLEMENTARY MATERIAL

Assessing the accuracy of magnetic resonance imaging in identifying early rectal cancers suitable for endoscopic intermuscular dissection

Lisa van der Schee, Rachel Carten, Sander C. Albers, Janneke van den Bergh, Manon N. G. J. A. Braat, Arnold C. Goede, Sabrine Kol, Miangela M. Lacle, Banafsche Mearadji, Shira I. Moos, Irene M. Nota, Nicky H. G. M. Peters, Jip F. Prince, Jorik J. Reimerink, Arantza Fariña Sarasqueta, Jeanette van Vooren, Jan Hein T. M. van Waesberghe, Barbara A. J. Bastiaansen, Frank P. Vleggaar, Leon M. G. Moons, Karin Horsthuis, Gina Brown

Methods

Study variables

Patient details: including age, sex, height, weight, BMI, ASA and/or WHO performance scores, along with a brief past medical history, at the time of initial treatment.

Endoscopy details: date of, and reason for, endoscopy. Details of the rectal lesion as stated in the initial report, including location, size, morphology and granularity along with the presence or absence of a depression, spontaneous bleeding and/or non-lifting sign. Formal endoscopic classifications including: Paris classification, Kudo's Pit pattern and Vascular pattern (JNET classification).

Initial radiology details: Details obtained from the initial MRI radiology report included distance from the anorectal junction, size and morphology of the lesion, where stated. Initial staging including clinical T- and N- stage, presence or absence of EMVI, Tumour Deposits and assessment of CRM status.

Treatment details: Pre-treatment MDT date and suggested treatment/outcome. Details of any endoscopic or surgical resection including date, type of treatment (e.g. ESD, EID, TAMIS), whether an en bloc resection was achieved and whether any further treatment was required after initial resection.

Pathology Reports: Size and morphology of the lesion, along with complete pathological staging details in case of malignancy. Full staging data included pathological T- and N-stage, grade of tumour differentiation, presence/absence of lymphovascular invasion and high-grade tumour budding. The distance of closest invasion related to deep and lateral margins was recorded, along with pathological R status (R0/1/2), as stated in original initial reports.

The PRESERVE staging assessment method

Here we briefly report the step-wise method of assessment for determining the depth of tumour invasion in Early Rectal Cancers that was taught to the study radiologists**.**

1. Split the viewing screen in two, with sagittal images displayed in one window, and oblique axial images displayed alongside them in a second window.
2. Adjust image contrast and window to ensure imaging is displayed with the muscularis propria and pelvic floor muscles appearing black in colour.
3. Identify the suspected lesion. Once identified, use the locator tool to ensure both sagittal and oblique axial images are aligned at the centre of the lesion, which will equate to the most invasive border of the tumour. Zoom in on the lesion.
4. Identify the muscularis and submucosal layers of the normal bowel wall in an area adjacent to the tumour. The muscularis should appear black in colour, and the submucosa has a striped lighter grey appearance (Supplemental Figure 1).
5. Identify whether invasion into the muscularis layer can be identified at the centre of the lesion on at least 2 imaging sequences, using the sagittal, oblique axial and coronal images. Measure depth of invasion.
6. Identify whether invasion into the submucosal layer can be identified at the centre of the lesion on at least 2 imaging sequences, using the sagittal, oblique axial and coronal images. Measure depth of invasion.
7. Complete T-staging assessment according to the measurements above, using criteria below:

**MRI assessment features**	**MRI T-staging**
No macroscopic evidence of submucosal invasion	T0 (benign)
≥1 mm macroscopically visible spared submucosa *and* fully intact muscularis propria	T1sm1/sm2
<1 mm submucosa preserved with ≥1 mm macroscopically intact fibres of muscularis propria	T1sm3/$T2_{circ}$
<1 mm submucosa preserved with <1 mm macroscopically intact fibres of muscularis propria, *or* invasion through muscularis propria.	$\geq T2_{long}$

8. Assess other features to complete staging, eg. CRM status, nodal status, presence of EMVI or tumour deposits.

Results

Table 1s Characteristics of participating study radiologists.

Variable	Study radiologists<br>N = 12
Experience as radiologist, median (IQR), years	9.0 (8.75)
Experience as abdominal radiologist, median (IQR), years	5.5 (8.75)
Type of hospital, n (%)	
Academic	12 (100)
Non-academic	0 (0.0)
Average number of reported rectal cancer MRIs per month, n (%)	
1 – 4	2 (16.7)
5 – 9	6 (50.0)
10 – 14	3 (25.0)
15 – 20	0 (0.0)
21 – 25	0 (0.0)
26 – 30	1 (8.3)
Number of reported rectal cancer MRIs (in total), n (%)	
101 – 200	4 (33.3)
201 – 300	1 (8.3)
301 – 400	1 (8.3)
401 – 500	3 (25.0)
501 – 749	1 (8.3)
750 – 1000	2 (16.7)

Table 2s Diagnostic accuracy of individual study radiologists' MRI assessment as to whether a rectal lesion was locally resectable in the intermuscular plane.

Study radiologist	Agreement with histology (diagnostic accuracy)
Radiologist 1	68.3%
Radiologist 2	81.0%
Radiologist 3	78.1%
Radiologist 4	94.9%
Radiologist 5	77.8%
Radiologist 6	67.5%
Radiologist 7	77.3%
Radiologist 8	65.9%
Radiologist 9	64.3%
Radiologist 10	81.1%
Radiologist 11	78.1%
Radiologist 12	72.7%

Table 3s Technical details of MRI scans used in the study, per sequence.

Scan parameter	Sequence		
	Sagittal	Coronal	Axial
Voxel, median (range), mm^3	0.87 (0.34–3.52)	0.93 (0.25–3.08)	1.17 (0.18–3.40)
Slice thickness, median (range), mm	3 (3–5)	3 (1–4)	3 (2–4)
Number of signal averages (NSA), median (range)	2 (1–5)	1 (1–4)	1 (1–4)
Echo time (TE), median (range), ms	89 (60–163)	83 (56–136)	80 (55–153)
Repetition time (TR), median (range), ms	3700 (2500–9850)	2820 (1291–9383)	3957 (1259–10914)

Table 4s Association between patient and lesion characteristics and MRI consensus misdiagnosis as to whether a lesion is locally resectable in the intermuscular plane or not.

Variable	Misclassified N/total within group (%)	OR (95% CI)
Age, in years	-	1.01 (0.98 – 1.04)
Sex		
Female	13/71 (18.3)	Ref
Male	33/174 (19.0)	1.04 (0.52 – 2.19)
ASA		
ASA I	9/44 (20.5)	Ref
ASA II	29/168 (17.3)	0.81 (0.36 – 1.96)
ASA III	8/33 (24.2)	1.24 (0.41 – 3.70)
BMI	-	0.94 (0.86 – 1.02)
Location*		
Proximal rectum	8/45 (17.8)	Ref
Mid rectum	12/56 (21.4)	1.26 (0.47 – 3.53)
Distal rectum	26/138 (18.8)	1.07 (0.46 – 2.72)
Lesion size*, in mm	-	1.01 (0.99 – 1.03)
pT-stage		
Adenoma	3/18 (16.7)	Ref
T1	12/110 (10.9)	0.61 (0.17 – 2.91)
T2	23/77 (29.9)	2.13 (0.63 – 9.82)
T3 or more	8/40 (20.0)	1.25 (0.31 – 6.32)

* Location and size of the lesion as assessed during endoscopy
ASA = American Society of Anesthesiologists; BMI= body mass index; CI= confidence interval; OR= odds ratio; Ref= reference category

Fig. 1s A pT2_{circ} shows loss of visible submucosa (yellow) on MRI with >1mm preserved muscularis propria (red) in the central portion of this early T2 tumour (blue). An example of a pT1sm2 cancer shows a continuous layer of >1mm visible submucosa (yellow) with >1mm preserved muscularis propria (red) seen at the central portion of this T1 tumor (blue). A hyper-intense (white) mucin layer highlights the external surface of the tumor.

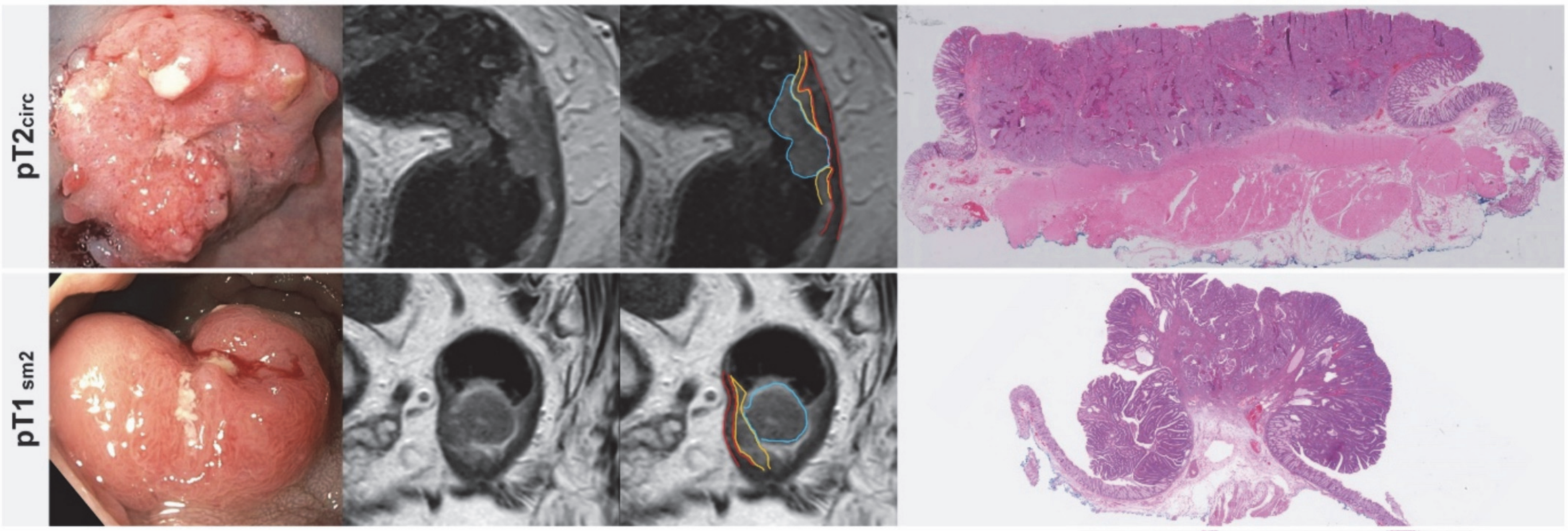

Supplement: Supplementary file 1 — Supplementary Material [file 10-1055-a-2621-2515_26415723.pdf]
